# Effects of Different Trehalose and Sorbitol Impregnation Methods on Freeze–Thaw Damage to Potato Slices

**DOI:** 10.3390/foods14132389

**Published:** 2025-07-06

**Authors:** Wenfang Xuan, Yiyang Qi, Xueqian Wan, Xuemei Gao, Haiou Wang, Huichang Wu

**Affiliations:** 1 College of Tea and Food Technology, Jiangsu Vocational College of Agriculture and Forestry, Zhenjiang 212400, China; xuanwf0617@163.com; 2 School of Food Science, Nanjing Xiaozhuang University, Nanjing 211171, China; qyy196439@163.com (Y.Q.); wxq050810@163.com (X.W.); who1978@163.com (H.W.); 3 Nanjing Institute of Agricultural Mechanization, Ministry of Agriculture and Rural Affairs, Nanjing 210014, China; gaoxuemei@caas.cn

**Keywords:** potato, trehalose, sorbitol, freeze–thaw damage, impregnation method

## Abstract

Fresh-cut potato slices are prone to browning. Although freezing is an effective method of preserving food, freezing and thawing cause inevitable damage to potato tissues. This study explored the freeze-protective effects of trehalose and sorbitol under atmospheric pressure impregnation and vacuum impregnation by analyzing their influences on the cell structural and textural characteristics of frozen–thawed potato slices. The results showed that both trehalose and sorbitol can significantly improve the quality of frozen–thawed potato slices. Vacuum impregnation resulted in a higher total sugar content in the impregnated potato slices than atmospheric pressure impregnation (*p* < 0.05). Sorbitol impregnation significantly reduced cell damage and nutrient loss of frozen–thawed potato slices; specifically, under vacuum impregnation conditions, the juice loss rate and relative electrical conductivity decreased to 7.58 ± 0.47% and 32.90 ± 1.83 mS/cm, respectively. Texture analysis showed that sorbitol impregnation resulted in significantly higher puncture hardness and TPA hardness in frozen–thawed potato slices than trehalose impregnation. Furthermore, observations of cell activity and transmission electron microscopy of potato tissues verified sorbitol’s advantages in maintaining cell structure integrity and reducing ice crystal damage. Hence, sorbitol vacuum impregnation is highly recommended as a pretreatment in potato quick freezing processes. This study provides a theoretical basis and technical support for the improvement of the quality of quick-frozen potato products, and for the later processing and manufacturing of frozen potato slices.

## 1. Introduction

As the world’s fourth most important staple crop following rice, wheat, and corn, potatoes hold significant agricultural and nutritional value. Their global prominence stems from a unique combination of attributes: they are notable for being rich in carbohydrates while also containing appreciable amounts of proteins, lipids, and essential vitamins, offering exceptional dietary flexibility. The tuber’s agricultural advantages are equally remarkable, demonstrating robust environmental adaptability, short growth cycles, high yield potential per cultivation unit, and versatile processing applications. These characteristics collectively account for their widespread cultivation and consumption across diverse geographical regions. However, their post-harvest preservation presents challenges, as stored tubers are susceptible to microbial contamination and biochemical degradation of nutritional components, which inevitably compromises product quality over time [1].

In recent years, freezing has been extensively employed as a preservation method for perishable and seasonal foods due to its remarkable efficacy. This preservation strategy enjoys widespread acceptance among consumers, the majority of whom hold the belief that it exerts minimal adverse effects on the nutritional quality of food [2].

The mechanism of freezing technology lies in the integration of low-temperature preservation advantages and the crystallization of water molecules into ice. However, the process of ice crystal formation, which encompasses both the size and location of the crystals, may lead to the disruption of cell membranes. This can result in a high degree of drip loss upon thawing and the leaching of nutrients [3]. Therefore, ensuring adequate low-temperature protection of frozen foods is of paramount importance in enhancing the physical and chemical properties of foods that have undergone freezing and thawing processes. However, traditional freezing methods have some drawbacks, such as slow freezing speed and the formation of large and uneven ice crystals, which can compromise food quality, resulting in a decline in quality after thawing. Therefore, improving freezing technology has become key to enhancing the quality of frozen foods. A new trend in the research field involves combining new freezing technologies with antifreeze agents as an innovative technology to regulate the formation of ice crystals.

Cryoprotectants—including sugars and chitosan—are frequently utilized to suppress ice recrystallization, preventing the growth of ice crystals that can result in mechanical damage to food tissues [4]. As cryoprotectants, sugars exhibit stabilizing effects on the stress tolerance of biological systems. Their hydrophilicity enables them to stabilize cell structures through hydrogen bonding [5]. Consequently, sugar impregnation can enhance the quality of frozen fruits and vegetables [6]. Polyhydroxy carbohydrate compounds are widely used in the processing of fruits and vegetables due to their strong hydration properties, good permeability to tissue cells, and excellent food processing attributes. Trehalose and sorbitol can effectively protect the natural flavors and nutrients in food and protect proteins and cells from damage caused by ice crystals [7]. Velickova et al. demonstrated that strawberries impregnated with a combined solution of trehalose and antifreeze proteins significantly improved their freeze resistance [5]. Similarly, Chen et al. found that treating kiwifruit with a trehalose solution helped to maintain the flavor and texture of the thawed fruit [7]. Dermesonlouoglou et al. reported that osmotic pretreatment with fructooligosaccharides and maltodextrin effectively preserved the color, hardness, and tissue integrity of quick-frozen strawberries [8]. Trehalose and sorbitol are widely used cryoprotectants; however, further investigation is needed to determine the effects of different impregnation methods on freeze–thaw damage in potato slices.

This study employs fresh-cut potato tissues as experimental objects to comparatively evaluate the cryoprotective efficacy of sorbitol and trehalose solutions under atmospheric pressure impregnation and vacuum impregnation treatments, focusing on the differences in quality indicators, microstructure, and cell activity of the frozen–thawed potato tissues. The effects of trehalose and sorbitol under different impregnation methods are evaluated in order to enhance their cryoprotective effects on potato tissues. The findings of this study provide a technical and theoretical basis for improving the quality of frozen potato products.

## 2. Materials and Methods

### 2.1. Materials and Regents

Fresh potatoes (Kexin No. 1, Inner Mongolia, China) were purchased from Nanjing Suguo Supermarket. For each experiment, a single batch of fresh potato samples was collected within 3 days of harvesting. The potatoes were meticulously selected to ensure the absence of mechanical damage (analyzed using sensory detection) and uniformity in size (the lengths and weights of the purchased potatoes were measured using a ruler; they were also weighed to ensure uniformity in size), and were stored at 4 °C in a refrigerator prior to use (but for no more than 3 days). Trehalose and sorbitol (both of food grade) were supplied by Shijiazhuang Ruixue Pharmaceutical Co., Ltd. (Shijiazhuang, China), which was sealed in a package and stored in a refrigerator at 4 °C. Trypan blue (0.4%) was obtained from Shanghai Macklin Biochemical Co., Ltd. (Shanghai, China), and the FAA fixative solution (formalin–acetic acid–alcohol, purity ≥ 99%) was provided by Wuhan Saivil Biotechnology Co., Ltd. (Wuhan, China). The reagents such as ethanol, phenol, concentrated sulfuric acid, paraffin, and periodic acid–Schiff reagent were all purchased from Tianjin Kemio Chemical Reagent Co., Ltd. (Tianjing, China).

### 2.2. Instruments and Equipment

The H0008-SMS texture analyzer was supplied by Beijing Yingsheng Hengtai Technology Co., Ltd. (Beijing, China). The L6S UV–visible spectrophotometer was manufactured by Shanghai Kexiao Scientific Instruments Co., Ltd. (Shanghai, China).The H1650 centrifuge was manufactured by Changsha Xiangyi Centrifuge Instrument Co., Ltd. (Changsha, China). The LRH-150 incubator was manufactured by Shanghai Huitai Instrument Manufacturing Co., Ltd. (Shanghai, China). The PHS-3E –40 °C freezer was manufactured by Shanghai Yidian Scientific Instrument Co., Ltd. (Shanghai, China).The ME104 analytical balance was manufactured by Shenzhen Shanxingsheng Electronic Technology Co., Ltd. (Shenzhen, China). The FE38-Standard conductivity meter was manufactured by Mettler-Toledo Instruments Co., Ltd. (Shanghai, China). Lastly, the SHZ-DIII corrosion-resistant circulating water vacuum pump was manufactured by Shanghai Tengfang Instrument Equipment Co., Ltd. (Shanghai, China).

### 2.3. Experimental Methods

#### 2.3.1. Preparation of Potato Slice Samples Impregnated with Trehalose or Sorbitol Solution

The fresh potatoes were washed and moisture was removed from their surfaces. Then, they were sliced into round discs with a thickness of 5 mm and a diameter of approximately 20 mm. The discs were placed in Erlenmeyer flasks, and a 10% trehalose or sorbitol solution (prepared using distilled water) was added at a material-to-solution ratio of 1:4 (mass ratio). Based on our previous experiments, two impregnation methods were employed: atmospheric pressure impregnation for 0.5 h, 1 h, 1.5 h, 2 h, and 2.5 h, and vacuum impregnation for 30 min, 40 min, 50 min, 60 min, and 70 min, during which the vacuum impregnation pressure was maintained at 0.05 MPa. After treatment, the potato slices were carefully removed, and the sugar solutions were drained from their surfaces using filter paper. The samples were then frozen at –40 °C for 24 h, followed by thawing at 25 °C for 2 h in a constant-temperature incubator. For each condition, three parallel experiments were set up.

#### 2.3.2. Total Sugar Content Determination

The total sugar content was determined based on the method developed by Uddin et al., with minor modifications [9]. A 1 g sample was weighed and placed in a mortar, and 5 mL of 80% ethanol was added. The sample was ground into a homogeneous slurry and transferred into a 10 mL centrifuge tube. The tube was placed in an 80 °C water bath and heated for 30 min with occasional stirring to ensure thorough extraction. Following heating, the tube was centrifuged at 10,000 r/min for 10 min to separate the supernatant, which was collected and diluted to 25 mL with deionized water to prepare the solution for soluble sugar measurements. To prepare the standard glucose solution, 1 g of glucose was accurately weighed and dissolved in distilled water, and then diluted to a final volume of 100 mL. This solution was further diluted to achieve a concentration of 3 mg/mL. Standard glucose solutions with volumes of 0.0, 0.1, 0.2, 0.3, 0.4, 0.5, and 0.6 mL were each diluted to 2.0 mL with distilled water. To each of these solutions, 1.0 mL of 6% phenol solution was added and mixed, followed by 5.0 mL of concentrated sulfuric acid. After standing for 30 min and cooling to room temperature, the absorbance of each solution was measured at 490 nm, with distilled water as a blank reference. A standard curve was constructed by plotting glucose concentration on the x-axis against absorbance on the y-axis. For the sample solution, 2 mL was taken and subjected to the same treatment as the standard solutions. The total sugar content was determined based on the constructed glucose standard curve. Each sample was analyzed in triplicate, and the results were averaged to ensure accuracy and reliability. The total sugar content was determined using the following equation:

Total sugar content (mg/g) = (C × N × V)/m

where C is the concentration from the standard curve (mg/mL), N is the dilution factor, V is the final volume of sample solution (mL), and m is the mass of the sample (g).

#### 2.3.3. Juice Loss Rate

The Juice loss rate of the thawed samples was determined using a method adapted from Sun et al. [10]. The potato samples were weighed (m_1_) after the impregnation treatment. After freezing treatment, the frozen samples were thawed at 25 °C for 2 h, and then reweighed (m_2_) after removing the liquid from their surfaces [11]. The juice loss rate was calculated as follows:

Juice loss rate/% = (m_1_ − m_2_)/m_1_ × 100

#### 2.3.4. Relative Electrical Conductivity Measurement

The relative electrical conductivity of the thawed samples was measured using a method by Wang et al., with a few modifications [12]. After thawing, the frozen potato slices were rinsed with deionized water. Two slices were placed in a 100 mL beaker with 50 mL of deionized water, and the initial conductivity, E_0_, was measured. Then, the beaker was sealed with plastic wrap and incubated for 1 h, after which the conductivity, E_1_, was measured again. The beaker was then boiled for 20 min, cooled to room temperature, and topped up to the original volume with deionized water to measure conductivity E_2_. Relative electrical conductivity was calculated as follows:

E = (E_1_ − E_0_)/E_2_ × 100

where E is the relative electrical conductivity (%); E_0_ is the initial conductivity after thawing (mS/cm); E_1_ is the conductivity after 1 h (mS/cm); and E_2_ is the conductivity after boiling (mS/cm).

#### 2.3.5. Puncture Measurement

After freeze–thaw treatment, puncture tests were performed using a texture analyzer. The “puncture-1000N” program with P/5 probe was used. Parameters: initial force 0.5 N, puncture depth 2 mm, return distance 20 mm, and test speed 60 mm/min. Each sample was tested four times, and the results were averaged.

#### 2.3.6. TPA Measurement

The texture profile analysis (TPA) of the frozen–thawed potato slices was performed using the TPA-1000N program with a 50 mm probe. Parameters: trigger force 0.5 N, starting height 20 mm, deformation 30%, and speed 60 mm/min. Four replicates were performed per group and the average value was taken.

#### 2.3.7. Observation of Cell Activity

Cell activity was determined using the method proposed by Zhang et al., with slight modifications [13]. Frozen–thawed potato samples were sliced to a thickness of less than 1 mm. The slices were stained with 0.4% Trypan blue for 2 min, and then placed in decolorizing solution with replacement every 5 h until the solution turned pale blue or colorless. The stained slices were spread flat on a white light board for image collection.

#### 2.3.8. Structural Analysis Using Light Microscopy (LM)

Potato slices (10 × 10 × 5 mm) were fixed in a formalin–acetic acid–alcohol solution (FAA, 90% ethanol, 5% acetic acid, and 5% formalin, *v*/*v*/*v*) for 24 h, embedded in paraffin, and sliced. Slices were stained with periodic acid–Schiff (PAS), dehydrated, and finally sealed on glass slides for optical microscopy. The CaseViewer 2.4 software was used to capture the original images of PAS-stained potato slices at the same magnification. The Image J 1.8.0 software was used to convert the original images to grayscale. The grayscale images were binarized to identify the edge contours of the cells. Then, intracellular impurity elimination was performed on the binarized expression image. Next, the average cell cross-sectional area (*A*) and cell cross-sectional circumference (*L*) of the final images were calculated using Image J 1.8.0 software. The equivalent diameter (*D*) was calculated using the formula below:

$$D=\sqrt{\frac{4A}{\pi }}$$

where *D* represents the equivalent diameter of the cell cross-section (μm) and A represents the cross-sectional area of the cell (μm^2^).

Cell roundness (*S_R_*): The degree to which the cross-section of a cell approaches a theoretical circle. SR was computed using the formula below:

$$S_{R}=\frac{4\pi A}{L^{2}}$$

where *S_R_* represents the roundness of the cell cross-section, A represents the cell cross-sectional area (μm^2^), and *L* represents the cell cross-section circumference (μm).

#### 2.3.9. Structural Analysis Using Transmission Electron Microscopy (TEM)

All TEM tests were conducted at 25 °C based on the method proposed by Wang et al., with some modifications [14]. Potato tissues were cut into 10 × 10 × 5 mm pieces, fixed in 3.5% glutaraldehyde in phosphate buffer, then rinsed with 0.1 mol/L of phosphate-buffered saline (PBS, pH 7.4), and post-fixed in 1% osmium tetroxide (OsO_4_), followed by another PBS rinse. The samples were dehydrated using an ethanol gradient (30%, 50%, 70%, 80%, 95%, and 100%), transitioned with epoxy propane, embedded in Spurr resin, and polymerized at 60 °C for 48 h. Sections were prepared with an LKB ultramicrotome and then stained. The sections were observed under a JEOL JEM-1400 transmission electron microscope (JEOL Inc., Tokyo, Japan) at 7000 × magnification.

### 2.4. Statistical Analysis

The experimental data were processed and graphs were generated using Excel 2019, Design-Expert 10, and OriginPro 2016. Data are presented as mean ± standard error of the mean (SEM). One-way analysis of variance (ANOVA) was executed using IBM SPSS Statistics 22.0 (IBM Corp, New York, NY, USA). The samples in each group were drawn from a normally distributed population, and the variances of all groups were equal. Statistical comparisons were performed using the Tukey–Kramer test (*p* < 0.05).

## 3. Results and Discussion

### 3.1. Total Sugar Content of Potato Slices After Impregnation

Determination of total sugar content after impregnation reflects the effectiveness of sugar penetration into the tissue. As shown in Table 1, compared with the control group (no impregnation), both impregnation methods significantly increased the total sugar content in potato slices, with vacuum impregnation showing greater effectiveness. This enhancement is likely due to the pressure differential in vacuum impregnation, which facilitates the penetration of the sugar solution. Consequently, vacuum impregnation offers advantages such as faster diffusion, more uniform distribution, and improved nutrient retention, as indicated by the higher total sugar content observed [15].

Under atmospheric pressure impregnation conditions, the total sugar content of potato slices treated with trehalose initially increased and then decreased over time. The highest total sugar content was observed at 2 h, which was significantly higher than at other time points (*p* < 0.05). In contrast, for sorbitol-treated samples, the total sugar content exhibited a fluctuating trend, reaching a peak at 1.5 h, which was also significantly higher than at other time points (*p* < 0.05). Under vacuum impregnation conditions, the total sugar content of the trehalose-treated group peaked at 60 min (354.63 ± 11.17), showing no significant difference compared to 50 min, but was significantly higher than at other time points (*p* < 0.05). For sorbitol treatment, the highest total sugar content was observed at 50 min (500.80 ± 13.76), which was significantly higher than at other time points (*p* < 0.05). The extended impregnation time led to a decrease in the total sugar concentration. It might be reasoned that the excessive impregnation time caused a significant decrease in the content of endogenous soluble sugars, raw pectin, and total pectin in the potato tissue due to reverse infiltration migration in sugar solution [16]. At the same time, the excess vacuum impregnation time probably caused a certain degree of damage to the potato tissue cells, resulting in a decrease in the total sugar content.

Comparison of the optimal time points for the two impregnation methods showed that the optimal time for trehalose treatment under atmospheric pressure was 2 h (337.17 ± 4.15), whereas that for vacuum impregnation was 60 min (354.63 ± 11.17), indicating that vacuum impregnation achieved higher total sugar content in a shorter time. For sorbitol, the optimal impregnation time under atmospheric pressure was 1.5 h (365.83 ± 9.35), and under vacuum conditions was 50 min (500.80 ± 13.76), again showing that vacuum impregnation resulted in a higher sugar content in less time.

In terms of the effect of the two sugars, sorbitol resulted in higher total sugar content under most impregnation conditions. For instance, under atmospheric impregnation for 1.5 h, sorbitol (365.83 ± 9.35) was significantly higher than trehalose (268.47 ± 7.80) (*p* < 0.05); under vacuum impregnation for 50 min, sorbitol (500.80 ± 13.76) was also significantly higher than trehalose (345.17 ± 7.05) (*p* < 0.05). Taiwo K A et al. [17] found similar results. The relative molecular mass of sorbitol is small, and the greater the molecular weight of the solute in the solution, the lower the osmotic pressure of the solution, and the smaller the mass transfer rate. The osmotic pressure of the freezing solution in the sorbitol treatment group is relatively high, resulting in more solutes entering the interior of the potato slice tissue, thereby increasing the total sugar content.

In summary, vacuum impregnation combined with sorbitol treatment can significantly increase the total sugar content of potato slices within a short time. The optimal condition is vacuum impregnation for 50 min, during which the total sugar content under sorbitol treatment reached the highest value of 500.80 ± 13.76 mg/mL. According to Table 1, vacuum impregnation effectively increases the soluble sugar content in potatoes and helps shorten the freezing time. This finding is similar to those reported by [18] on changes in the content of soluble solids in vacuum-impregnated apples. The increase in the content of soluble solids can reduce the likelihood of recrystallization in potato slices during freeze–thaw cycles, proving that trehalose and sorbitol are effective cryoprotectants. Some sugar molecules enter the potato cells during impregnation, enhancing the water retention capacity of fruits and vegetables.

### 3.2. Juice Loss Rate of Frozen–Thawed Potato Slices

The juice loss rate is a key indicator for measuring the extent of cellular damage caused by freezing. During thawing, the water from melted ice crystals cannot be fully reabsorbed by the potato tissue cells, accelerating the juice loss rate and leading to nutrient loss and changes in cell structure [19]. The juice loss rate of frozen-thawed potatoes under the two impregnation methods is shown in Table 2.

Under both atmospheric and vacuum impregnation conditions, the juice loss rate of potato slices treated with trehalose at different impregnation times showed no significant difference compared with the control group. However, in the sorbitol treatment group, the juice loss rates of all potato slices were significantly decreased compared with the control group. Compared with trehalose, sorbitol has a better protective effect on potato slices. Sorbitol has a protective effect on the cell membrane during the freezing process as it can replace water by combining with the polar groups of phospholipids, thereby maintaining the integrity of the membrane structure. It increases the water-holding capacity of potatoes during freezing and storage to a certain extent, effectively reducing the juice loss rate of potatoes [20]. This is also associated with the freezing rate of potato slices using different sugar solutions. Compared with trehalose, sorbitol has a smaller molecular weight, resulting in faster heat transfer, thereby increasing the freezing rate. It can penetrate potato cells more quickly during the impregnation process, increasing the probability of nucleation within the cells and generating finer ice crystals, enhancing the water-holding capacity of fruits and vegetables, thus reducing the rate of juice loss.

Comparing the optimal impregnation times under the two methods showed that for trehalose treatment, the optimal treatment time under atmospheric pressure was 2 h (12.83 ± 0.81), whereas under vacuum impregnation, it was 60 min (11.40 ± 0.94), indicating that vacuum impregnation reached a lower juice loss rate in a shorter time. For sorbitol, the optimal treatment time under atmospheric impregnation was 1.5 h (7.80 ± 0.67), whereas under vacuum it was 70 min (7.58 ± 0.47), showing little difference between the two methods. Tappi et al.’s research on the juicing of fresh-cut melons also revealed that vacuum juicing can better preserve the texture of the raw materials during storage, despite the high variability in the raw materials [21].

In terms of the effects of the two sugars, sorbitol showed lower juice loss rates under most impregnation conditions. For example, under atmospheric impregnation for 1.5 h, sorbitol (7.80 ± 0.67) had a significantly lower juice loss rate than trehalose (12.92 ± 0.45) (*p* < 0.05); additionally, under vacuum impregnation for 70 min, sorbitol (7.58 ± 0.47) had a significantly lower juice loss rate than trehalose (12.55 ± 0.19) (*p* < 0.05). This may be due to some sorbitol molecules entering the potato cells during impregnation, making sorbitol a better cryoprotectant. It can effectively maintain the structural integrity of potato cells, reduce damage to the cell wall by ice crystals, and enhance the water-holding capacity of fruits and vegetables, thereby reducing the juice loss rate [22].

### 3.3. Relative Electrical Conductivity of Frozen–Thawed Potato Slices

Conductivity can be used to indicate the degree of cell membrane damage; the higher the value, the higher the permeability of the cell membrane and the lower its functional integrity [23]. By measuring the conductivity of potato samples after different impregnation treatments, the extent of tissue damage in frozen–thawed potato slices can be evaluated. Table 3 shows the results of conductivity changes in frozen–thawed potato slices under different treatments.

Under atmospheric impregnation conditions, the conductivity of the trehalose treatment group showed no significant change and no significant difference compared with the control group. In contrast, the conductivity of the sorbitol treatment group was lowest at 1.5 h (31.87 ± 1.46), which was significantly lower than at other time points (*p* < 0.05). Under vacuum impregnation, the conductivity of the trehalose treatment group showed a significant downward trend, with the lowest value at 70 min (24.91 ± 0.57), which is significantly lower than at other time points (*p* < 0.05). The sorbitol treatment group exhibited the same trend, with the lowest conductivity at 70 min (32.90 ± 1.83), which was also significantly lower than other time points (*p* < 0.05).

Comparing the optimal time points under the two impregnation methods showed that the optimal time for trehalose treatment under atmospheric impregnation was 2 h (38.08 ± 0.18), whereas under vacuum it was 70 min (24.91 ± 0.57), showing that vacuum impregnation reached a lower conductivity in a shorter time. For sorbitol, the optimal time under atmospheric impregnation was 1.5 h (31.87 ± 1.46), whereas under vacuum it was 70 min (32.90 ± 1.83), indicating little difference between the two methods.

In terms of the effect of the two sugars, at 1.5 h of atmospheric impregnation, sorbitol (31.87 ± 1.46) was significantly lower than trehalose (38.55 ± 2.98) (*p* < 0.05); at 70 min of vacuum impregnation, trehalose (24.91 ± 0.57) was significantly lower than sorbitol (32.90 ± 1.83) (*p* < 0.05). Under atmospheric conditions, sorbitol treatment resulted in relatively lower conductivity, whereas under vacuum conditions, trehalose showed a lower conductivity.

Both sugar solution impregnations effectively reduced the conductivity of frozen–thawed potato slices and decreased tissue damage. Except for the 30 and 40 min vacuum groups, the conductivity of the sorbitol impregnation group was significantly lower than that of the control group. Moreover, except at 1 h, the conductivity in the atmospheric sorbitol group was also significantly lower than in the trehalose group. This indicates that sorbitol provides better protection to cells during freezing, reducing electrolyte leakage, which is consistent with the results of total sugar content and juice loss rate [24].

### 3.4. Puncture Hardness of Frozen–Thawed Potato Slices

**Figure 1:**
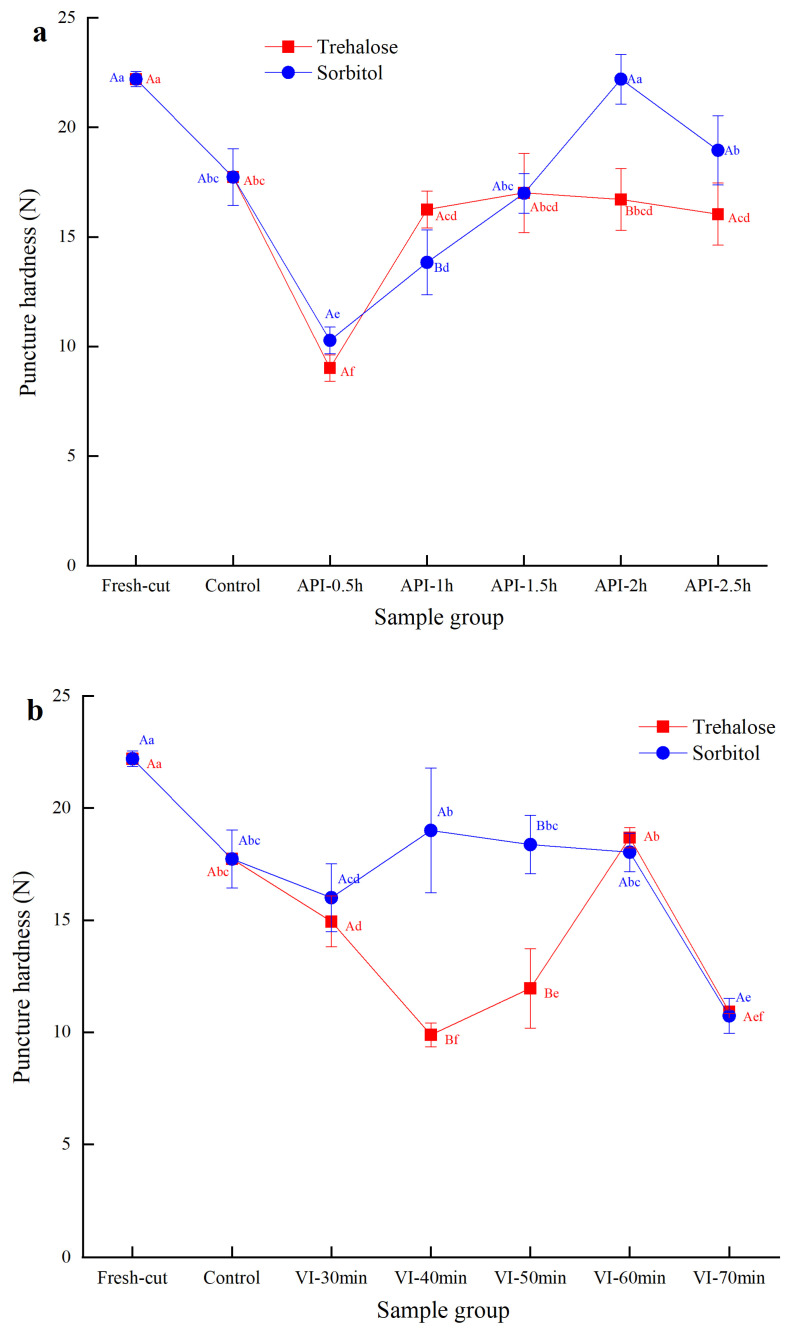
Puncture hardness of the frozen–thawed potato slices. (**a**) Atmospheric pressure impregnation method; (**b**) vacuum impregnation method; Fresh-cut represents fresh-cut samples; Control represents samples with no impregnation; API represents atmospheric pressure impregnation; and VI represents vacuum impregnation. Different uppercase letters indicate significant differences in puncture hardness between different sugar solution treatment groups; different lowercase letters indicate significant differences between different impregnation treatment groups.

The puncture method is widely used for evaluating the texture of fruits and vegetables. This method is not limited by the size or shape of the sample being tested; it only requires puncturing at a specific part of the specimen, thereby significantly reducing errors in sample pretreatment. Based on Figure 1, compared with fresh-cut samples, the hardness of frozen–thawed samples in both control groups was significantly lower (*p* < 0.05), possibly due to the formation of ice crystals during refreezing that caused cell lysis. The tissue softened significantly after thawing, resulting in decreased hardness [25].

Figure 1 indicates that the puncture hardness shows a trend of first decreasing and then increasing, and subsequently decreasing again with the extension of impregnation time. Under atmospheric pressure impregnation, the puncture hardness of potato slices treated with trehalose was highest at 1.5 h, reaching 17.01 ± 1.81, which was close to the non-impregnated control group. For sorbitol treatment, the highest hardness was reached at 2 h with a value of 22.20 ± 1.13, which was significantly higher than at other time points (*p* < 0.05) and close to the untreated group. Under vacuum impregnation, the highest puncture hardness for trehalose treatment was 18.68 ± 0.45 at 60 min, whereas for the sorbitol-treated group, the highest hardness was 19.00 ± 2.78 at 40 min; both values were higher than those of the non-impregnated control group. This phenomenon was also observed in cherries [26].

Comparison of the optimal treatment times between the two impregnation methods indicated that the optimal time for trehalose treatment under atmospheric pressure was 1.5 h (17.01 ± 1.81), whereas under vacuum conditions, it was 60 min (18.68 ± 0.45), indicating that vacuum impregnation had a shorter optimal time and resulted in higher puncture hardness. For sorbitol, the optimal treatment time under atmospheric pressure was 2 h (22.20 ± 1.13), whereas under vacuum, it was 40 min (19.00 ± 2.78). Under these conditions, atmospheric impregnation achieved higher puncture hardness, while vacuum impregnation showed a smaller difference in hardness but significantly reduced treatment time. These findings are similar to those reported by Jha et al. on the quality of melon and apple tissues [27].

In terms of sugar type, sorbitol exhibited higher puncture hardness under both impregnation conditions. At 2 h under atmospheric pressure, sorbitol (22.20 ± 1.13) resulted in significantly higher puncture hardness than trehalose (16.71 ± 1.41) (*p* < 0.05); additionally, at 40 min under vacuum, sorbitol resulted in significantly higher puncture hardness (19.00 ± 2.78) than trehalose (9.88 ± 0.53) (*p* < 0.05). Except for the sorbitol group treated for 2 h under atmospheric pressure, the hardness of the other sugar-treated groups was significantly lower than that of the untreated group, although most showed no significant difference compared with the control. This is likely because the interaction between sugar solutions and water minimizes ice crystal damage to the potato cell structure and improves water retention capacity [28], thereby enhancing hardness.

### 3.5. TPA Texture Characteristic Parameters of Frozen–Thawed Potato Slices

The effects of different impregnation treatments on the TPA hardness, chewiness, cohesiveness, and springiness of freeze-dried potato slices are shown in Table 4. Hardness is the most important texture quality of frozen potato products after thawing, affecting consumer acceptance. In the trehalose-treated group under atmospheric pressure impregnation, TPA results showed no significant difference in hardness compared with the control group (*p* > 0.05), and, except for the 70 min treatment group, vacuum impregnation also showed no significant difference from the control group (*p* > 0.05). The hardness in the sorbitol-treated group was significantly lower than that in the trehalose-treated group.

The chewiness index of samples treated for different impregnation durations was also significantly lower than that of the fresh group, with trehalose treatment showing better results than the sorbitol group. In terms of cohesiveness, sugar-treated samples showed a significant increase compared with the fresh group, and there was little difference between the trehalose and sorbitol treatments. Most treatment groups showed no significant differences (*p* > 0.05). Regarding springiness, the 70 min trehalose treatment under vacuum impregnation exhibited the lowest value.

Based on overall texture indicators, most of the composite indices in the sorbitol-treated group were lower than those in the control group, and the frozen–thawed group was lower than the fresh group, indicating that the potato tissue suffered some damage during the freeze–thaw process. The reason for these differences may be that during freezing, ice crystals form in the potatoes, and due to pressure differences, intracellular water moves outward, forming unevenly distributed ice crystals. These ice crystals damage the integrity of the potato cell walls and alter the structure of the potato tissue during thawing, causing ruptures between the cell membrane, cytoplasmic layer, and cell wall. The cellular contents flow out with the tissue fluid, resulting in a significant decrease in hardness and cohesiveness, and the tissue becomes more deformable [29]. Studies have found that excessive vacuum impregnation time can cause a certain degree of damage to the tissue cells of fruits and vegetables [30], affecting the product quality.

The actual effect of sorbitol impregnation on potatoes may depend on multiple factors, including the treatment time and impregnation conditions. Based on the comprehensive experimental indicators, it was found that the overall indices of vacuum-impregnated potato slices were relatively better than those of atmospheric pressure impregnation. The vacuum impregnation group treated for 60 min showed better texture and puncture hardness; therefore, this condition was selected for the observation of cell activity and microstructure.

### 3.6. Trypan Blue Staining of Cells in Frozen–Thawed Potato Slices

**Figure 2:**
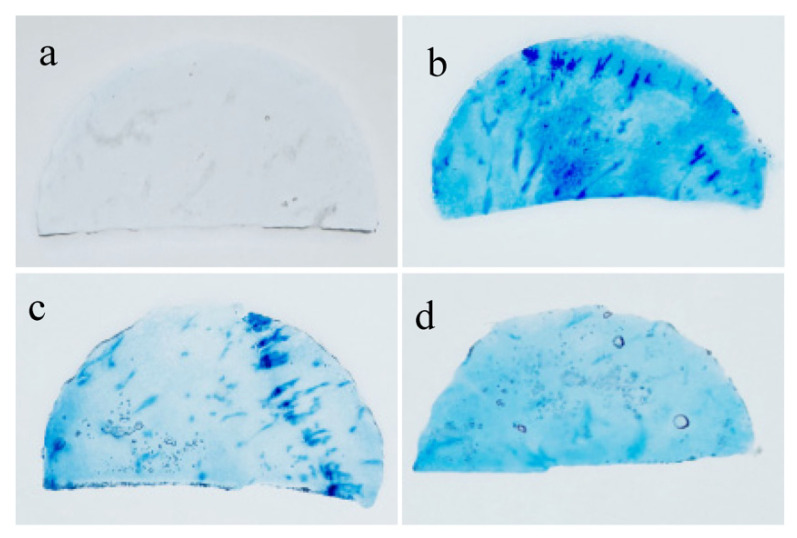
Images of cell viability staining of the frozen–thawed potato slices subjected to different treatments. (**a**) fresh-cut sample; (**b**) control sample (without impregnation); (**c**) vacuum impregnation with trehalose solution for 60 min; and (**d**) vacuum impregnation with sorbitol solution for 60 min.

Freezing-induced damage to potato cell membranes can lead to cell death. Trypan blue staining can penetrate necrotic cell membranes and stain the nuclei blue, serving as a qualitative indicator of the quantity of dead cells [31]. The results of the staining of potato slices subjected to different treatments are shown in Figure 2. A large number of living cells with intact membrane structures were present in fresh potato slices, which effectively excluded the Trypan blue solution, preventing the cells from being stained. In the control group, a large number of cells had lost activity and their cell membranes had lost selective permeability; this resulted in them becoming more permeable, leading to widespread staining by Trypan blue.

In potato slices treated with trehalose, only a small portion of cells lost viability, and only a small area was stained blue by the trypan blue solution. In contrast, the sorbitol-treated group showed the lightest coloration, with only a few dark blue spots observed, indicating few signs of cell death. Liu et al. obtained similar results following their analysis of the impact of sorbitol impregnation on freeze–thaw damage to different frozen potato slices [32]. This suggests that trehalose and sorbitol can replace water molecules and bind to polar residual hydrogen bonds, stabilizing the cell membrane, protecting protein structures, and reducing damage caused by dehydration during freezing, thereby greatly improving cell survival rates [33]. Thus, impregnation with trehalose and sorbitol solutions can effectively inhibit cell death in potatoes during the freeze–thaw process, with sorbitol showing better performance.

### 3.7. Optical Microscope Observation of PAS Staining of Frozen–Thawed Potato Slices

The periodic acid–Schiff (PAS) staining method involves a reaction between periodic acid (HIO_4_) and compounds containing ethylene glycol, which produce two aldehyde groups that then react with Schiff reagent to form a purplish-red substance. Glycogen granules appear purplish-red, and cell nuclei appear blue. The integrity of the cell wall and tissue structure significantly affects the texture of potato slices.

**Figure 3:**
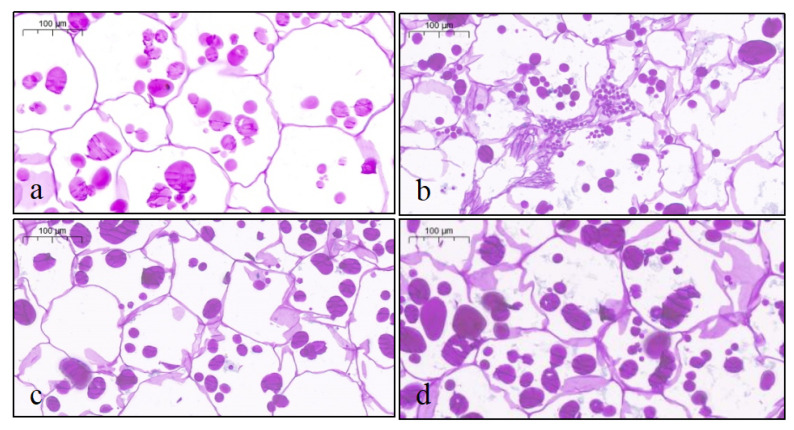
Optical microscope images (at 10× magnification) of the frozen–thawed potato slices subjected to different treatments. (**a**) Fresh-cut samples; (**b**) control samples (without impregnation); (**c**) vacuum impregnation with trehalose solution for 60 min; and (**d**) vacuum impregnation with sorbitol solution for 60 min.

In Figure 3, cells in fresh potato slices are tightly arranged, with complete, rounded structures and clearly defined contours. However, the freeze–thaw treatment caused noticeable structural changes in the cell walls of the potato slices in the control group, resulting in significant cell rupture and shrinkage. The cells appeared relatively flattened, and the original oval shape of the starch granules disappeared.

Although a few cells in the sugar-treated groups exhibited some structural shrinkage, the degree of deformation was minimal, and the cellular structures were well preserved. This is because vacuum-treated tissues exhibit higher cellular structural integrity [34]. These findings indicate that both sugar solution treatments effectively maintained the structural integrity of potato cells, reducing the damage caused by ice crystals to the cell wall and membrane.

Additionally, the sugar molecules can cross-link with cell wall components, increasing the thickness and strength of potato cell walls, thereby reducing susceptibility to rupture and maintaining integrity. A similar phenomenon was observed in strawberries [35]. In the trehalose-treated group, the purplish-red color was relatively lighter, indicating less polysaccharide degradation in the tissue. Sun et al. found that frozen potato slices soaked in trehalose had a minimal degree of deformation, and the cell structure remained well preserved, whereas the sorbitol-treated group formed more glycogen granules [10]. The integrity of the microstructure reflects the preservation of the natural shape and structure of frozen potato slices, suggesting a reduction in juice loss during the thawing process [36].

Freeze–thaw cycles caused significant deterioration of the cell structure of fresh-cut potatoes. The cell equivalent diameter and cell roundness of the cell structure in potato slices are shown in Table 5. Compared with the fresh samples (cell equivalent diameter: 36.138 ± 1.87 μm, circularity: 0.899 ± 0.04), the cell equivalent diameter of the untreated control group decreased sharply to 17.021 ± 1.82 μm, and the cell circularity decreased to 0.601 ± 0.03 (*p* < 0.05), confirming that ice crystal damage led to cell collapse and morphological distortion. Vacuum impregnation treatment significantly alleviated the above damage (*p* < 0.05). The cell equivalent diameters of the trehalose and sorbitol impregnation groups were significantly better than those of the control group, indicating that both were more advantageous in protecting the integrity of the cell structure.

### 3.8. Transmission Electron Microscopy of the Microstructure of Frozen–Thawed Potato Slices

**Figure 4:**
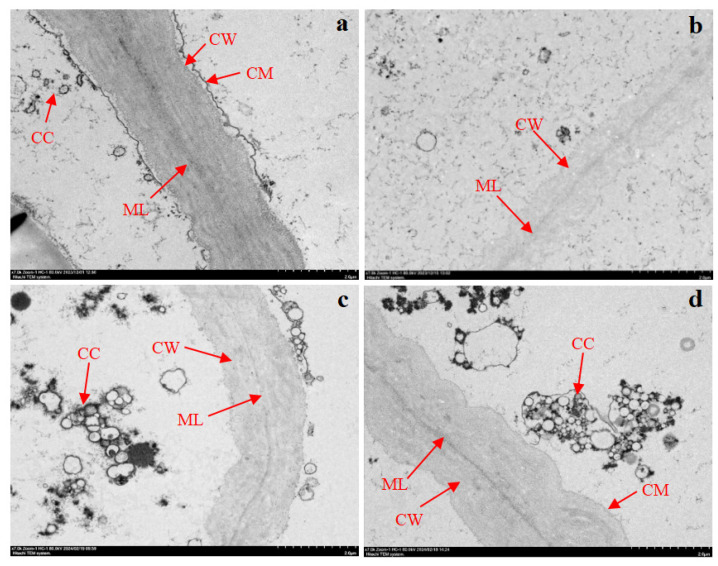
TEM images (at 7000 magnification) of the frozen–thawed potato slices subjected to different treatments. (**a**) Fresh-cut samples; (**b**) control samples (without impregnation); (**c**) vacuum impregnation with trehalose solution for 60 min; and (**d**) vacuum impregnation with sorbitol solution for 60 min. CW represents the cell wall; ML represents the middle lamella; CM represents the cell membrane; and CC represents the cellular content.

Figure 4 shows the TEM images of frozen–thawed potato slices subjected to different treatments. Initially, the cell wall and membrane structures of fresh potatoes appeared intact, with clear edges. The cell wall consisted of dense longitudinal fibrous structures and a middle lamella, with cytoplasm and cellular contents distributed around the cell wall.

No cell membranes were observed in the control group or the trehalose-treated group; additionally, all cellular contents were lost in the control group. In contrast, the sorbitol-treated samples exhibited relatively intact cell structures, with clearly visible cell membranes. Additionally, the cell walls in both sugar-treated groups were thicker than those in the control group, suggesting that trehalose and sorbitol can alter the thickness or rigidity of the cell wall by modifying substances such as lignin and cellulose [37].

Comparing Figures, Figure 4c,d show higher cell contents in the sorbitol group, with a more pronounced cell membrane and darker-colored cell wall and intermediate layer. The cell membrane disappears, and the color of the cell wall and intermediate layer is significantly lighter in Figure 4c.

## 4. Conclusions

This study involved a comprehensive analysis of the differences in physico-chemical properties, texture, and cellular structures of frozen–thawed potato slices under different sugar solution impregnation treatments, enabling a comparison of the cryoprotective effects of trehalose and sorbitol pretreatments. Both trehalose and sorbitol impregnation effectively improved the quality of frozen–thawed potato slices and protected the cell structure. The total sugar concentration of potato slices in the sorbitol treatment group was significantly higher than that in other treatment groups at 50 min, reaching 500.80 mg/mL. Freeze–thaw damage to the potato tissues was lowest (*p* < 0.05) when the potato slices were impregnated under vacuum for 50–70 min, and puncture hardness was lowest at 70 min of vacuum impregnation (10.73 ± 0.78). Texture analysis and histological morphology observation showed that sorbitol vacuum impregnation pretreatment had a better effect. The addition of both sugar solutions enhanced the water retention capacity of the samples, which helped to improve cell structure and reduce freezing-induced damage. In particular, potato samples treated with sorbitol vacuum impregnation for 60 min exhibited the least freeze–thaw damage due to the protective effects of impregnation.

In summary, it is feasible to use trehalose and sorbitol solution pretreatments for freezing potatoes. This approach holds practical significance in addressing the problem of cellular structure damage in quick-frozen potato products during freeze–thaw cycles, thereby extending their shelf life and improving nutritional value. It can also provide high-quality raw materials for the post-production and processing of potato slices, and offers a theoretical reference for the processing and preservation of pre-made potato slices. However, this study did not include in-depth investigations of the synergistic protective effects of different sugar solutions on potato slices, the mechanisms underlying the protective effects of different sugars, or whether there is an improvement effect on the size and position of ice crystals during the impregnation process. In future research, atomic force microscopy will be used to detect the effects of sugars on the degradation of pectin substances during the freezing of fruits and vegetables, measure membrane permeability, and explore whether sugar can inhibit the damage caused by ice crystals to cell membranes.

## Figures and Tables

**Table 1:** The total sugar content (mg/g).

Impregnation Method	Impregnation Time	Trehalose	Sorbitol
Control (without impregnation)		61.07 ± 7.38 ^Af^	61.07 ± 0.37 ^Ai^
Atmospheric pressure impregnation	0.5 h	217.93 ± 14.64 ^Be^	284.17 ± 5.19 ^Af^
	1 h	222.63 ± 12.74 ^Be^	237.13 ± 6.77 ^Ah^
	1.5 h	268.47 ± 7.80 ^Ad^	365.83 ± 9.35 ^Ac^
	2 h	337.17 ± 4.15 ^Aab^	259.27 ± 9.90 ^Bg^
	2.5 h	299.57 ± 10.62 ^Ac^	311.17 ± 9.11 ^Ae^
Vacuum impregnation	30 min	332.83 ± 5.80 ^Bb^	423.60 ± 12.61 ^Ab^
	40 min	333.17 ± 14.67 ^Bb^	357.83 ± 4.46 ^Ac^
	50 min	345.17 ± 7.05 ^Bab^	500.80 ± 13.76 ^Aa^
	60 min	354.63 ± 11.17 ^Aa^	352.13 ± 7.04 ^Ac^
	70 min	335.37 ± 5.41 ^Ab^	335.47 ± 7.60 ^Ad^

Notes: Different uppercase letters indicate significant differences in total sugar content among different sugar solution treatment groups. Different lowercase letters indicate significant differences among the different impregnation treatment groups.

**Table 2:** The juice loss rate of the frozen-thawed potato slices (%).

Impregnation Method	Impregnation Time	Trehalose	Sorbitol
Control (without impregnation)		12.57 ± 0.55 ^Aab^	12.57 ± 0.55 ^Aa^
Atmospheric pressure impregnation	0.5 h	13.07 ± 2.15 ^Aab^	8.01 ± 1.58 ^Bd^
	1 h	13.26 ± 0.54 ^Aa^	8.22 ± 0.91 ^Bd^
	1.5 h	12.92 ± 0.45 ^Aab^	7.80 ± 0.67 ^Bd^
	2 h	12.83 ± 0.81 ^Aab^	8.80 ± 0.85 ^Bcd^
	2.5 h	13.51 ± 0.65 ^Aa^	10.60 ± 0.51 ^Bb^
Vacuum impregnation	30 min	12.31 ± 0.99 ^Aab^	8.51 ± 0.24 ^Bcd^
	40 min	12.39 ± 0.02 ^Aab^	9.73 ± 0.27 ^Bbc^
	50 min	12.23 ± 0.98 ^Aab^	8.18 ± 1.06 ^Bd^
	60 min	11.40 ± 0.94 ^Ab^	7.69 ± 0.69 ^Bd^
	70 min	12.55 ± 0.19 ^Aab^	7.58 ± 0.47 ^Bd^

Notes: Different uppercase letters indicate significant differences in juice loss rate among different sugar solution treatment groups. Different lowercase letters indicate significant differences among the different impregnation treatment groups.

**Table 3:** Relative electrical conductivity of the frozen–thawed potato slices (%).

Impregnation Method	Impregnation Time	Trehalose	Sorbitol
Control (without impregnation)		41.66 ± 2.06 ^Aabc^	41.66 ± 2.06 ^Ab^
Atmospheric pressure impregnation	0.5 h	41.92 ± 2.75 ^Aabc^	37.59 ± 1.27 ^Bc^
	1 h	38.77 ± 1.58 ^Abcd^	38.50 ± 3.37 ^Ac^
	1.5 h	38.55 ± 2.98 ^Abcd^	31.87 ± 1.46 ^Be^
	2 h	38.08 ± 0.18 ^Abcd^	33.63 ± 1.49 ^Bde^
	2.5 h	48.85 ± 2.15 ^Aa^	35.86 ± 0.80 ^Bcd^
Vacuum impregnation	30 min	45.52 ± 0.93 ^Aab^	41.63 ± 1.41 ^Bb^
	40 min	36.94 ± 1.20 ^Bbcd^	45.21 ± 1.26 ^Aa^
	50 min	33.63 ± 1.38 ^Bcde^	37.61 ± 1.18 ^Ac^
	60 min	31.44 ± 1.35 ^Bde^	36.05 ± 1.75 ^Acd^
	70 min	24.91 ± 0.57 ^Be^	32.90 ± 1.83 ^Ade^

Notes: Different uppercase letters indicate significant differences in relative electrical conductivity among different sugar solution treatment groups. Different lowercase letters indicate significant differences among the different impregnation treatment groups.

**Table 4:** TPA texture characteristic parameters of the frozen–thawed potato slices.

Impregnation Method	Impregnation Time	Hardness/N		Chewiness/N·mm		Cohesiveness/Ratio		Springiness/mm	
		Trehalose	Sorbitol	Trehalose	Sorbitol	Trehalose	Sorbitol	Trehalose	Sorbitol
Fresh-cut samples		188.25 ± 2.86 ^Aa^	188.25 ± 2.86 ^Aa^	94.06 ± 1.54 ^Aa^	94.06 ± 1.54 ^Aa^	0.42 ± 0.01 ^Ab^	0.42 ± 0.01 ^Ad^	1.19 ± 0.01 ^Aa^	1.19 ± 0.01 ^Aa^
Control (without impregnation)		43.36 ± 4.46 ^Ab^	43.36 ± 4.46 ^Ab^	21.88 ± 2.39 ^Abc^	21.88 ± 2.39 ^Ab^	0.52 ± 0.01 ^Aab^	0.52 ± 0.01 ^Aabc^	0.97 ± 0.02 ^Abcd^	0.97 ± 0.02 ^Ab^
Atmospheric pressure impregnation	0.5 h	37.19 ± 0.68 ^Ab^	21.25 ± 0.38 ^Bghj^	17.60 ± 6.03 ^Abc^	8.48 ± 1.22 ^Bf^	0.50 ± 0.07 ^Ab^	0.51 ± 0.04 ^Aabc^	0.92 ± 0.19 ^Abcd^	0.77 ± 0.04 ^Be^
	1 h	39.60 ± 3.69 ^Ab^	25.31 ± 2.00 ^Bdefg^	17.90 ± 1.71 ^Abc^	10.36 ± 1.86 ^Bef^	0.48 ± 0.02 ^Ab^	0.50 ± 0.02 ^Abc^	0.95 ± 0.02 ^Abcd^	0.82 ± 0.06 ^Bde^
	1.5 h	36.84 ± 4.69 ^Ab^	27.87 ± 2.05 ^Bdef^	16.29 ± 0.28 ^Ac^	14.04 ± 1.22 ^Bcde^	0.51 ± 0.02 ^Ba^	0.58 ± 0.02 ^Aa^	0.87 ± 0.06 ^Ade^	0.87 ± 0.05 ^Abcde^
	2 h	40.10 ± 1.93 ^Ab^	19.85 ± 0.54 ^Bi^	21.88 ± 2.29 ^Abc^	8.57 ± 0.67 ^Bf^	0.53 ± 0.07 ^Aab^	0.51 ± 0.02 ^Aabc^	1.02 ± 0.02 ^Ab^	0.84 ± 0.07 ^Bcde^
	2.5 h	44.57 ± 3.66 ^Ab^	29.07 ± 3.36 ^Bde^	16.68 ± 4.55 ^Ac^	12.17 ± 3.60 ^Acdef^	0.68 ± 0.28 ^Aa^	0.52 ± 0.05 ^Babc^	0.87 ± 0.05 ^Ade^	0.80 ± 0.06 ^Ade^
Vacuum impregnation	30 min	42.35 ± 10.68 ^b^	37.04 ± 3.99 ^c^	18.89 ± 5.7 ^3bc^	15.87 ± 0.98 ^cd^	0.49 ± 0.01 ^b^	0.49 ± 0.03 ^c^	0.89 ± 0.05 ^Acde^	0.87 ± 0.02 ^Abcde^
	40 min	38.24 ± 7.01 ^b^	29.77 ± 1.88 ^d^	17.89 ± 3.52 ^bc^	16.81 ± 1.86 ^c^	0.52 ± 0.03 ^ab^	0.57 ± 0.03 ^ab^	0.91 ± 0.05 ^Abcd^	0.99 ± 0.03 ^Ab^
	50 min	37.64 ± 4.77 ^b^	24.81 ± 3.16 ^efg^	18.64 ± 0.95 ^bc^	12.46 ± 2.51 ^cdef^	0.54 ± 0.03 ^ab^	0.54 ± 0.05 ^abc^	0.92 ± 0.02 ^Abcd^	0.92 ± 0.02 ^Abcd^
	60 min	45.46 ± 7.18 ^b^	33.98 ± 1.69 ^c^	23.76 ± 2.41 ^b^	14.82 ± 3.56 ^cde^	0.52 ± 0.02 ^ab^	0.49 ± 0.08 ^c^	1.00 ± 0.07 ^Abc^	0.89 ± 0.08 ^Abcde^
	70 min	22.06 ± 2.05 ^c^	23.41 ± 2.71 ^fgi^	8.48 ± 1.71 ^d^	12.85 ± 4.10 ^cdef^	0.49 ± 0.04 ^b^	0.57 ± 0.05 ^ab^	0.77 ± 0.03 ^Ae^	0.95 ± 0.15 ^Abc^

Notes: Different uppercase letters indicate significant differences in TPA texture characteristic parameters among different sugar solution treatment groups. Different lowercase letters indicate significant differences among the different impregnation treatment groups. The chewiness index is calculated as follows: Chewiness = Hardness × Cohesiveness × Springiness.

**Table 5:** Cell structure parameters of the potato slices subjected to different treatments.

Impregnation Method	Cell Equivalent Diameter/(μm)	Cell Roundness
Fresh-cut samples	36.138 ± 1.87 ^a^	0.899 ± 0.04 ^a^
Control (without impregnation)	17.021 ± 1.82 ^c^	0.601 ± 0.03 ^d^
Vacuum impregnation with trehalose solution for 60 min	23.422 ± 1.37 ^b^	0.658 ± 0.05 ^c^
Vacuum impregnation with sorbitol solution for 60 min.	20.891 ± 1.75 ^b^	0.716 ± 0.04 ^b^

Note: Different lowercase letters in the same column indicate significant differences among the different impregnation treatment groups (p < 0.05).

## Data Availability

The original contributions presented in the study are included in the article. Further inquiries can be directed to the corresponding author.
